# Development of genomic resources for the narrow-leafed lupin (*Lupinus angustifolius*): construction of a bacterial artificial chromosome (BAC) library and BAC-end sequencing

**DOI:** 10.1186/1471-2164-12-521

**Published:** 2011-10-21

**Authors:** Ling-Ling Gao, James K Hane, Lars G Kamphuis, Rhonda Foley, Bu-Jun Shi, Craig A Atkins, Karam B Singh

**Affiliations:** 1 Plant Industry, Commonwealth Scientific and Industrial Research Organisation, Private Bag No. 5, Wembley WA 6913, Australia; 2 Centre for Food and Genomic Medicine, Perth WA 6000, Australia; 3 Australian Centre for Plant Functional Genomics, University of Adelaide, Glen Osmond SA 5064, Australia; 4 School of Plant Biology, The University of Western Australia, Crawley WA 6009, Australia; 5 The Institute of Agriculture, The University of Western Australia, Crawley WA 6009, Australia

## Abstract

**Background:**

*Lupinus angustifolius *L, also known as narrow-leafed lupin (NLL), is becoming an important grain legume crop that is valuable for sustainable farming and is becoming recognised as a potential human health food. Recent interest is being directed at NLL to improve grain production, disease and pest management and health benefits of the grain. However, studies have been hindered by a lack of extensive genomic resources for the species.

**Results:**

A NLL BAC library was constructed consisting of 111,360 clones with an average insert size of 99.7 Kbp from *cv *Tanjil. The library has approximately 12 × genome coverage. Both ends of 9600 randomly selected BAC clones were sequenced to generate 13985 BAC end-sequences (BESs), covering approximately 1% of the NLL genome. These BESs permitted a preliminary characterisation of the NLL genome such as organisation and composition, with the BESs having approximately 39% G:C content, 16.6% repetitive DNA and 5.4% putative gene-encoding regions. From the BESs 9966 simple sequence repeat (SSR) motifs were identified and some of these are shown to be potential markers.

**Conclusions:**

The NLL BAC library and BAC-end sequences are powerful resources for genetic and genomic research on lupin. These resources will provide a robust platform for future high-resolution mapping, map-based cloning, comparative genomics and assembly of whole-genome sequencing data for the species.

## Background

The genus *Lupinus*, belongs to the subfamily Papilionoideae of the Leguminosae (syn. Fabaceae) forming an important part of Papilionoideae, occupying the genistoid clade which is phylogenetically distinct from its sister clades. These sister clades contain the majority of scientifically and economically important legumes: Aeschynomnoid-Dalbergioids (*Arachis *[peanut]), Phaseoloid-Millettioids (*Glycine *max [soybean]) and *Phaseolus vulgaris *[common bean]) and the Hologalegina clade (*Lotus japonicus, Medicago truncatula, Pisum sativa *[pea], *Vicia faba *[broad bean], *Lens culinaris *[lentil], *Trifolium *[clover] and *Cicer arietinum *[chickpea]). In the past decade, concerted efforts have been directed to understanding the genomics, evolution and biological characterisation of these sister clades [1-4]. However, genistoids are the least exploited group of legumes and lupins remain as one of the lesser studied legume crops.

Lupins have traditionally been used for animal feed but are gaining recognition as a health food for humans, due to their unique dietary composition. The seeds contain a high level of protein (30-40%) and dietary fibre (30%), low oil and negligible starch, resulting in the lowest recorded Glycaemic Index of any commercial grain crop [5]. The protein content in lupin seed is similar to that in soybean, but lupin has lower levels of phytoestrogens which may potentially constitute a significant health risk [6,7]. Thus, lupins are an attractive alternative to soybean. Moreover, lupin seeds also contain constituents that alter satiety and other features of human health with the prospect of pharmaceutical potential [8-10]. With increased incidence of obesity and the associated risk of diabetes and cardiovascular disease, lupins are an excellent candidate as a healthy food.

Lupins are adapted to a range of highly divergent climatic and environmental conditions [11], providing direct and indirect benefits in rotation with cereal crops under rain fed conditions and limited soil nutrient supply. Despite their agronomic potential lupins are not widely exploited and, as a consequence, have not attracted the intense molecular research required for genomic characterisation. In contrast, a wealth of genomic resources has been generated for two model legumes, *Medicago truncatula *and *Lotus japonicus *[2,3]. In recent years, genetic and genomic resources have also been developed to different degrees for other major grain and pasture legume crops, including pea (*Pisum sativum*), soybean (*Glycine max *L. Merr.), common bean (*Phaseolus vulgaris *L.), mung bean (*Vigna radiate*), chickpea (*Cicer arietinum *L.), cowpea (*Vigna unguiculata*), pigeon pea (*Cajanus cajan *L), groundnut (*Arachis hypogaea *L.) and clover (*Trifolium repens *L) [12-18]. For lupin, the major significant advances have been the establishment of draft genetic maps for both NLL and white lupin (*Lupinus albus *L), which employed a variety of techniques and markers. The majority of the markers were derived from comparative genomics among related legume and non-legume species [19-24]. Also in white lupin candidate genes and proteins associated with antimicrobial defense and heavy-metal uptake have been identified using genomic and proteomic approaches [25] as have a large number of proteins, transcripts and microRNAs present in the phloem translocation stream [26]. For NLL, a limited number of ESTs and genomic sequences have been submitted to the NCBI databases, and a narrow-leafed lupin BAC library from *cv *Sonet has been constructed with 6 × coverage of the genome [27]. Nevertheless, to facilitate the fast growing research activities in lupins, additional genomic and genetic resources are needed. The genomic and genetic resources can also benefit comparative genomic studies with other legumes.

BAC libraries and BAC-end sequences are valuable resources, which have contributed significantly to genetic and genomic studies of a wide range of model or economically important plant species (see review [12]). Using BAC-end sequencing, a large number of SSR markers have been identified which have in turn provided tools for developing the genetic and physical maps of legumes such as *G. max, T*. *repens *L, *C. arietinum *and *C. cajan *[13,14,16,17,28-31]. BAC-end sequences have permitted identification of macro- and micro-synteny between species and have provided accurate and cost-effective means to estimate genome properties such as genome organisation and composition of some legume crops [17,30,32].

This study reports the construction and characterisation of a nuclear-genome BAC library of the NLL *cv*. Tanjil. *cv*. Tanjil was one of the parents used for two recombinant inbred line (RIL) populations that segregated for important domestication traits such as early flowering, bitterness (alkaloid production), pod shattering, water permeability of seed and resistance to anthracnose [20,33-35]. These populations have been important for development of molecular markers and genetic maps for the species. Moreover, *cv*. Tanjil has been chosen as the reference genome to be sequenced in a genome sequencing project that commenced in 2011. The BAC library for this cultivar was constructed using the *Bam*HI restriction enzyme and was successfully used to obtain BAC clones containing different members of the major seed storage protein, β-conglutin, family. In order to help determine the structure and composition of the NLL genome, 9600 randomly selected BAC clones were sequenced generating 13985 BAC end-sequences (BESs). Based on estimates that the genome size of NLL is 924 Mbp [36,37], these BESs covered approximately 1% of the genome. The NLL BESs provided useful information on the genome composition and organisation of the NLL genome. We also used the BESs to identify 9966 SSR motifs, some of which were shown to be potential molecular markers.

## Results

### Construction of a BAC library from NLL *cv *Tanjil

A NLL *cv*. Tanjil BAC library was constructed from hydroponically grown seedlings using the *Bam*H1 restriction enzyme. The library comprised of 111,360 BAC clones which were stored in 292 384-well microtitre plates. The average insert size of the library was approximately 99.7 kb based on the analysis of ca. 250 randomly selected clones. Approximately 2% of the clones contained no insert while the majority (ca. 75%) had insert sizes of between 90 kb and 110 kb, 12.5% above 110 kb, 9.2% between 80 kb and 90 kb, 3% between 50 kb and 80 kb and 0.3% smaller than 50 kb. The coverage of the library was estimated to be around 12 haploid genome equivalents according to the (haploid) genome size estimate of approximately 924 Mbp for NLL [36,37].

### Screening the BAC library for β-conglutin containing clones

To demonstrate the utility of the library, 111,360 BAC clones which were double-spotted onto six nylon membrane filters using a robot were screened for NLL β-conglutin genes using a cDNA fragment from a β-conglutin gene (*BETA2*) as the probe [38]. This cDNA probe cross-hybridised to the other six members of the β-conglutin gene family due to the high level of homology among the gene sequences [38]. The screening permitted 108 positive clones to be identified. Twelve clones were randomly selected and confirmed to contain β-conglutin gene(s) by PCR with a pair of primers able to recognise all seven β-conglutin gene members.

### Characterisation of β-conglutin containing BAC clones

**Figure 1:**
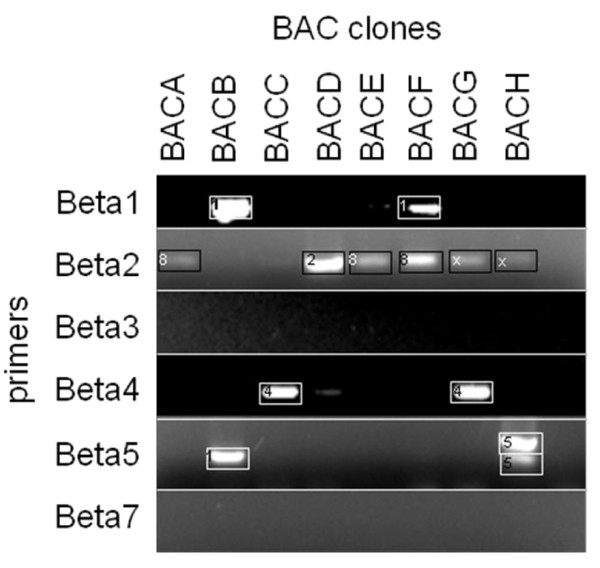
**PCR products and sequencing analysis of eight β-conglutin cross hybridising clones**. PCR products from eight BAC clones (BACA, BACB, BACC, BACD, BACE, BACF, BACG, BACH) using specific BETA1, 2, 3, 4, 5, and 7 primers and annealing temperatures [38], were separated on an agarose gel using electrophoresis. The PCR products (boxed) were sequenced and the best aligned β-conglutin sequence is numbered inside the box. 1 represents *BETA1*, 2 represents BETA2, 4 represents BETA4, 5 represents *BETA5*, 8 represents *BETA8*, and × represents bands for which no useable sequence data was obtained.

For further analysis, eight of the BAC clones were analysed for the presence of specific β-conglutin genes by PCR using primers and annealing temperatures specific for each β-conglutin gene, followed by sequencing of the PCR product. As shown in Figure 1, each of the eight β-conglutin hybridising BAC clones contained β-conglutin genes, including *BETA1, BETA2, BETA4, BETA5*, and a new β-conglutin gene, recognised by *BETA2 *primers, which has been renamed, *BETA8*. The truncated sequences of the eight β-conglutin genes are shown in Additional file 1. It is not clear why the *BETA5 *primers recognised *BETA1 *sequences from BACB but not from BACF. *BETA5 *primers detected two bands from BACH, however sequence data showed that they both contained *BETA5 *sequences, indicating a PCR aberration. *BETA1 *primers identified a product with sequence identity to *BETA1 *except there was an extra 111 bp, presumably an intron within BETA1. Apart from BACF, which contained *BETA1 *and *BETA8*, each BAC clone only contained one β-conglutin gene.

### Predicting characteristics of the NLL genome from bioinformatic analysis of a randomly sampled collection of BAC-end sequences

To provide insight into the sequence content and complexity of the NLL genome, we sequenced and analyzed both ends of 9600 BAC clones randomly selected from the library and generated a total of 17856 BAC-end sequences (BESs). Among these sequences, 13895 BESs (77.8%) of BAC-end sequences contained usable sequence data (> 100 bp after trimming) with a total length of ca. 8.89 Mbp which covers 0.96% of the NLL genome. The average length of the BESs was 683 bp with a maximum of 1112 bp and minimum of 101 bp. A total of 3961 of the BESs were discarded, because sequences were too short (< 100 bp), matched to the vector, showed low complexity or were identified as bacterial or organelle sequences.

Applying bioinformatic analyses to the 13985 high-quality BESs permitted prediction of various characteristics of the whole NLL genome. The overall G:C content of BESs was 39%. However, the G:C content within predicted gene-coding regions was higher at 45%. Within the 8.89 Mbp of BES sequence data, a total of 7014 repeats were identified representing 16.7% of the genome. Long terminal repeat (LTR) retrotransposons were the most abundant class of transposable elements, constituting 10.26% of the genome (Table 1). The most abundant LTR retroelements were Ty1/Copia-like repeats (6.14% of the genome), followed by Gypsy/DIRS1-like repeats (4.08%). Other repeats, including 461 simple repeats (0.28%), 3391 low complexity repeats (1.82%), 182 DNA transposons (0.46%) and 422 rDNA repeats (2.75%) were also identified.

**Table 1:** Repeat types within *L.angustifolius *BAC-end sequences identified with Repeatmasker (http://repeatmasker.org) versus REPBASE [57]

Repeat Type	Copy number	Total Length Occupied (bp)	% of Genome
**Retroelements**	**2551**	**1007849**	**11.34**
SINEs	9	761	0.01
LINEs	294	95086	1.07
RTE/Bov-B	67	13157	0.15
L1/CIN4	227	81929	0.92
LTR elements	2248	912002	10.26
Ty1/Copia	1296	545597	6.14
Gypsy/DIRS1	892	362514	4.08
**DNA transposons**	**182**	**40690**	**0.46**
Hobo-Activator	62	11795	0.13
Tc1-IS630-Pogo	1	117	0
En-Spm	48	14769	0.17
MuDR-IS905	17	1516	0.02
Tourist/Harbinger	35	6229	0.07
**Unclassified**	**7**	**596**	**0.01**
**Total Interspersed Repeats**		**1049135**	**11.81**
			
**Ribosomal RNA**	**422**	**244400**	**2.75**
**Simple repeats**	**461**	**25165**	**0.28**
**Low complexity sequence**	**3391**	**161502**	**1.82**

**Figure 2:**
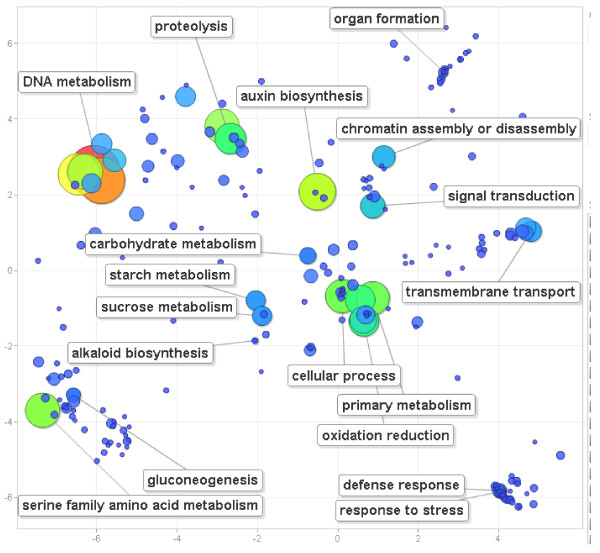
**Principal Component Analysis (PCA) scatter-plots (generated via REViGO **[60]) **of the abundance of gene ontology (GO) terms related to biological processes**. GO terms are represented by circles. Circles representing similar GO terms are clustered closer together than circles representing un-related GO terms. The sizes (large = high, small = low) and colours (red = high, green = moderate, blue = low) of circles are proportional to the numbers of functional annotations (GO terms) predicted in the BAC-end sequences of *Lupinus angustifolius*. GO terms representative of clusters of circles with high or low abundance have been labelled where appropriate.

To estimate the protein-encoding gene content of the NLL genome, the 13895 BESs were compared to the NCBI NR protein database by BLASTx. Protein-matching regions were found in 2667 BESs, 1723 of which also contained matches to known repetitive DNA sequences. Putative gene-encoding regions totalled 483,216 bp, equivalent to 5.4% of the total BES dataset. Based on this value and an estimated average gene length of 956 bp in legume species and 1170 bp over all plant species (source: http://www.phytozome.net) the NLL genome was predicted to contain between 42656 and 52204 genes. Blast2GO assigned 12831 Gene Ontology (GO) terms to 2930 BESs (Figure 2, Additional files 2 and 3). GO annotations were converted to 5448 FunCAT annotations assigned to 2067 BESs (Additional file 4).

Functional analysis of the encoded genes in the 13985 BESs predicted high proportions of the NLL gene content involved in the cell cycle and DNA processing (16.42%), protein binding (15.02%), protein folding and modification (5.75%), organelle proteins (4.76%) and metabolism (3.82%) (Additional files 5 and 6). A summary of gene ontology revealed a relatively higher proportion within the NLL BESs of genes associated with DNA metabolism, proteolysis, auxin biosynthesis, cellular processes, primary metabolism, oxidation and reduction and serine family amino acid metabolism (Figure 2). Conversely, there was a relative scarcity in the BESs of genes involved in organ formation, signal transduction, transmembrane transport, gluconeogenesis, alkaloid biosynthesis, starch and sucrose metabolism and defense/stress responses (Figure 2).

### Comparative genomics between NLL and other plant species

**Figure 3:**
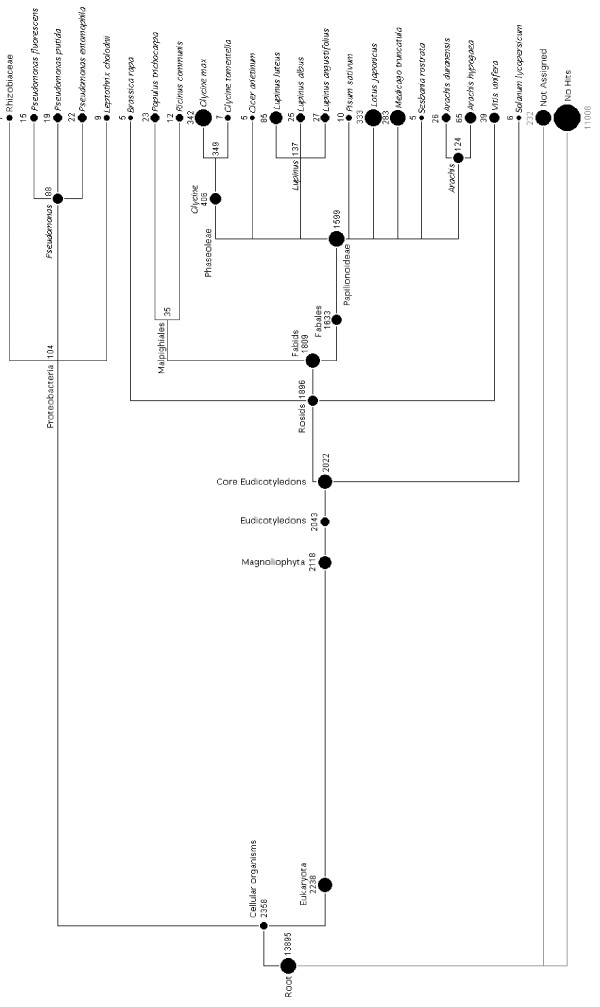
**Phylogenetic relationship of the 13895 BAC-end sequences (BESs) with the NCBI Nucleotide database**. The best hit to the NCBI Nucleotide database of each BES was determined by BLASTn [39] and the phylogenetic distribution of these hits was visualised with MEGAN [40]. Phylogenetic tree structure was derived from the NCBI taxonomy database. Circles represent taxons and their diameter is proportional to the number of BESs with an assigned hit. Numbers next to circles indicate the number of BESs inclusively mapped to a taxon (includes counts of daughter-taxons).

To examine the phylogenetic relationship between NLL and sequenced species, the 13895 quality-screened BESs were aligned (via BLASTn) to the NCBI Nucleotide database and the probable phylogenetic distribution of these hits was visualised with MEGAN [39,40] (Figure 3). MEGAN indicated the level of species similarity and sequence conservation within the randomly sampled BES subset of the NLL genome. About 18.3% of BESs matched sequences available in the NCBI Nucleotide database, while 79.2% of BESs had no hits. This is due in part to the relatively low number of sequences from *Lupinus *and other closely related genera that are currently available. The majority (84%) of the matched BESs were mapped to eudicotyledon species, 77.9% of which were assigned to the species of the subfamily Papilionoideae (Leguminosae). The three sequenced leguminous species, *G. max, L. japonicus *and *M. truncatula*, were highly represented followed by the genera *Lupinus *and *Arachis*. In contrast, there were only six NLL BESs that were best aligned to *Arabidopsis *sequences and no BESs specifically aligned to *Oryza sativa*. The average percent identity of BLASTn alignments between NLL BESs and *G. max, M. truncatula *and *L. japonicus *was 95.8%, 94.4% and 90.6% respectively. However the average identity restricted to predicted gene-encoded regions was 87.0%, 88.5% and 86.7% respectively. A small proportion of BESs (ca. 0.7%) were aligned to proteobacteria, perhaps due to the fact that the tissues for constructing the BAC library were collected from lupin seedlings grown under non-sterile conditions.

### Simple sequence repeat (SSR) profiling and SSR markers

To characterise NLL SSRs and compare them with those from *M. truncatula, G. max, L. japonicus*, Arabidopsis and *O. sativa*, SSRs were predicted using methods described previously [41]. A total of 9966 NLL SSR motifs were found in the 13895 BESs. Details of these SSRs, including the repeat length, repeat motif, and repeat period are presented in Additional file 7. The SSRs were divided into two classes: class I (≥ 20 bp) and class II (12-19 bp) [41]. The frequency of these SSR classes for all six species is compared in Table 2. Consistent with data for the other five species, class II SSRs in NLL were the most abundant microsatellites, being ca. 50 fold greater in frequency than class I SSRs. The individual frequencies of SSRs appeared to be lower in NLL than in the other five species. Based on the 8.89 Mbp of sequence (an estimated 1% of the NLL genome) obtained from the BES data, the average density for the class I (≥ 20 bp) SSRs was approximately one SSR every 0.9 Kbp which is slightly higher than one SSR every 0.6-0.7 Kbp for the other five species. For the class II SSRs (12-19 bp), the NLL SSRs appeared to be much less frequent and the average distance between SSRs was over six times that of soybean and three to four times that of the other four species.

**Table 2:** Frequency of class I (A) and class II (B) microsatellites per million base pairs in genomic sequences of six plant species (adapted from comparisons between five species performed [41])

A) Class I (≥ 20 bp total length)											
**Species**	**Total sequence length (Mbp)**	**G:C content**	**†Mono-**	**Di-**	**Tri-**	**Tetra-**	**Penta-**	**Hexa-**	**Hepta-**	**Octa-**	**Average distance (kbp)**

*Lupinus angustifolius*	8.89*	38.0	7.5	8.2	1	0.1	0.1	0.7	0.8	1.2	0.9 §
*Medicago truncatula*	77.13	33.4	19.7	37.5	9.5	4	6	2.4	3.1	0.7	0.6
*Glycine max*	20.15	36.0	6.1	63	43.2	3.5	3.9	1.4	7.4	0.3	0.7
*Lotus japonicus*	26.92	36.6	1.2	24	11.4	4.8	5.3	5.8	5.6	1	0.6
*Arabidopsis thaliana*	119.10	36.0	11.7	21.4	9.1	1.4	2.9	1.5	5.8	0.7	0.7
*Oryzae sativa*	474.66	43.5	2.3	29.8	13.9	7	7.9	3.7	2.4	0.8	0.6
											

**B) Class II (< 20 bp total length)**											

**Species**	**Total sequence length (Mbp)**	**G:C content**	**Mono-**	**Di-**	**Tri-**	**Tetra-**	**Penta-**	**Hexa-**	**Hepta-**	**Octa-**	**Average distance (Kbp)**

*Lupinus angustifolius*	8.89*	38.0	179.6	16.3	39.3	56.4	149.7	464.3	149.4	46.8	51.0 §
*Medicago truncatula*	77.13	33.4	184.2	43.7	98.8	128.2	44.1	812.7	270.6	88.1	12.1
*Glycine max*	20.15	36.0	75.9	52.4	96.5	119.9	31.4	749.4	256.7	73.5	7.8
*Lotus japonicus*	26.92	36.6	62.7	47	118.4	101.1	36.3	821.5	284.2	85.9	16.9
*Arabidopsis thaliana*	119.10	36.0	103	57.5	138	92.2	29.1	732.4	223.1	70.4	18.4
*Oryzae sativa*	474.66	43.5	44.8	69.3	204.7	127.6	37.9	804.1	232.6	85.3	14.7

* Total length of *L. angustifolius *BAC-end sequences

§ Estimate based on microsatellite frequencies and total length of *L. angustifolius *BAC-end sequences

†Mono-: mono-nucleotide repeats; Di-: di-nucleotide repeats; Tri-: tri-nucleotide repeats; Tetra-: tetra-nucleotide repeats;

Penta-: penta-nucleotide repeats, Texa-: texa- nucleotide repeats; Hepta-: hepta- nucleotide repeats; Octa-: octa- nucleotide repeats.

**Figure 4:**
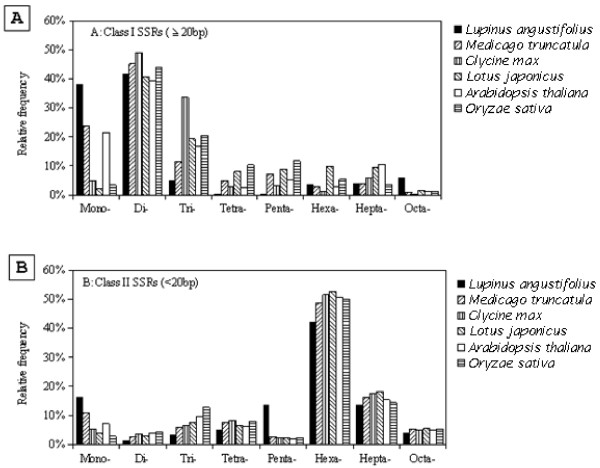
**Relative frequency of different motif length of SSRs**. A: Class I SSRs with lengths of 20 nucleotides or greater; B: Class II SSRs with lengths of 12 to 19 nucleotides. Mono-: mono-nucleotide repeats; Di-: di-nucleotide repeats; Tri-: tri-nucleotide repeats; Tetra-: tetra-nucleotide repeats; Penta-: penta-nucleotide repeats, Texa-: texa- nucleotide repeats; Hepta-: hepta- nucleotide repeats; Octa-: octa- nucleotide repeats.

However, analysis of the relative frequency of individual groups of SSRs with motif length 1-8 bp revealed some major trends for all six species as well as some distinctions for NLL SSRs (Figure 4). While the di-nucleotide SSRs in NLL were the most abundant of all the class I SSRs (≥ 20 nt), in line with the other 5 species, the mono- and octa-nucleotide SSRs showed higher representation and the tri-, tetra- and penta-nucleotide SSRs lower representation in NLL than those in other species. In contrast, the distribution of the relative abundance of each repeat motif length of the class II SSRs (12-19 nt) in NLL appeared consistent with that in the other species. Penta-nucleotide SSRs were an exception, and were relatively more abundant by about four times in NLL, than those of the other species. In the combination of both class I and class II SSRs, NLL appeared to have higher abundance of mono-nucleotide SSRs and lower abundance of tri- and tetra-nucleotide SSRs. In NLL, most of the penta-nucleotide SSRs had short repeat sequences and grouped into class II (12-19 bp).

To develop SSR markers for NLL, primer-pairs were designed flanking 2023 SSRs using Primer3 [42] with additional criteria described in the Materials and Methods. After taking repetitive sequences into account, there were 1497 non-redundant SSR marker candidates including 24 class I SSRs (≥ 20 bp) and 1455 class II SSRs (12-19 bp). These SSRs and the details of their primers are shown in Additional file 8. Twenty four additional class I SSR markers were designed using less stringent criteria (see Methods). The details of these 24 SSRs are shown in Additional file 9.

A subset of 24 Class I SSR primer pairs were used to establish a high throughput genetic mapping procedure for NLL, termed 'multiplex-ready PCR' (Hayden et al 2008), to determine the success rate of the identified SSRs as polymorphic markers between the parents of two NLL recombinant inbred line (RIL) populations. One RIL population was a narrow cross (*cv *Tanjil × *cv *Unicrop) and the other was a wide cross (P27255 "wild" × 83A:476 "domestic") [20,33]. The number of alleles that the primers amplified and their lengths are presented in Table 3. Of the 24 primer pairs tested, 3 did not yield any amplicons and four were monomorphic between all four NLL lines tested. The remaining 17 markers yielded single amplicons corresponding to their expected sizes based on the BES data. Two out of these 17 primer pairs also generated secondary amplicons for all four NLL lines. Interestingly, the wild NLL line P27255 had an additional two primer pairs that yielded multiple amplicons, whereas the other three domesticated NLL lines did not. Given that the BESs only cover 8.89 Mbp of the NLL lupin genome, the amplification of multiple products by a given primer pair could not be ruled out, but this appeared to be limited to only a few primer pairs.

**Table 3:** Overview of the length (in bp) of 24 class I SSR primers on the parents of two *L.angustifolius *recombinant inbred line populations (RILs). The length of the fragments presented is based on the length using the MRT primers which have 14 bp and 16 bp adaptors on the forward and reverse primers respectively

	Tanjil		Unicrop		83A:476 ("Domestic")		P27255 ("Wild")	
	Allele 1	Allele 2	Allele 1	Allele 2	Allele 1	Allele 2	Allele 1	Allele 2
LaSSR_001	278	-	278	-	278	-	264	-
LaSSR_002	243	-	243	-	243	-	226	-
LaSSR_003	324	-	326	-	324	-	343	-
LaSSR_004	323	-	323	-	323	-	323	-
LaSSR_005	325	-	327	-	325	-	344	-
LaSSR_006	No amplicon	-	No amplicon	-	No amplicon	-	No amplicon	-
LaSSR_007	293	-	293	-	293	-	293	-
LaSSR_008	318	-	318	-	284	-	281	-
LaSSR_009	328	-	328	-	328	-	300	-
LaSSR_010	298	221	277	233	298	233	298	221
LaSSR_011	344	-	348	-	348	-	348	301
LaSSR_012	246	-	249	-	249	-	245	205
LaSSR_013	231	-	231	-	231	-	231	
LaSSR_014	No amplicon	-	No amplicon	-	No amplicon	-	No amplicon	
LaSSR_015	321	282	289	282	321	282	326	282
LaSSR_016	No amplicon	-	No amplicon		No amplicon		No amplicon	
LaSSR_017	278	-	276	-	276	-	284	-
LaSSR_018	246	-	252	-	252	-	205	-
LaSSR_019	266	-	266	-	254	-	250	-
LaSSR_020	327	-	327	-	313	-	318	-
LaSSR_021	309	-	319	-	319	-	332	-
LaSSR_022	300	-	298	-	298	-	300	-
LaSSR_023	377	-	377	-	377	-	377	-
LaSSR_024	245	-	245	-	245	-	245	-

As expected the "wild" NLL line, P27255, was the most divergent, with the three domesticated lines being closely conserved (Table 3). Nine out of 24 markers were polymorphic between the parents of the narrow cross (*cv *Tanjil × *cv *Unicrop), whereas 14 markers were polymorphic between the parents of the wide cross (P27255 "wild" × 83A:476 "domestic").

## Discussion

A deep coverage and high quality BAC library was constructed for the NLL cultivar Tanjil, which is emerging as the reference genome for this species. The *cv*. Tanjil BAC library represents ca. 12 × haploid genome equivalents. It complements the previous NLL BAC library constructed using a different restriction enzyme (*BamH*1 vs *Hind*III) and a different NLL genotype (*cv*. Sonet) [27]. The library contains very low organelle contamination (0.02%). The quality of the library described here has been verified through the BAC-end sequencing of 9600 clones and the successful screening of the library with a probe for the NLL β-conglutin genes (Figure 1. 1). This NLL BAC library together with the pre-existing BAC library will help develop genetic and genomic tools for lupins and identify useful lupin genes for crop improvement and in relation to human health. Indeed, the screening of the NLL BAC library has resulted in the identification of a large number of BAC clones containing various types of β-conglutin genes (Figure 1). The NLL β-conglutins are potential lupin-specific allergens [38] and their further characterisation will verify their structures and functions, thereby ultimately helping reduce allergenicity problems, potentially through a genetic engineering approach.

The BAC-end sequencing of 9600 randomly selected BAC clones represents the initial phase of efforts to characterise the NLL genome. The BAC-end sequencing represents a random sampling of ca. 1% of the NLL genome. Therefore these BESs provide a preliminary genome-wide survey and facilitate comparisons with well-characterised legume and closely related non-legume species. The G:C content of NLL (39%) is slightly higher than estimates in related legume species: *P. Sativum *(37.7%), *G. max *(36%), *M. truncatula *(34%), *L. japonicus *(36%) and *Trifolium pratense *(34.2%) [41,43]. The BESs generated were sufficient to reconstruct and analyse the relative proportions of major repeat families. Based on the BESs obtained, the repetitive content in NLL is estimated to be at least 16.6% of the genome, which is close to *Lotus japonicus *(ca. 19%) and much lower than those of *M. truncatula *(ca. 38%) [44], soybean (ca. 59%) [13] and pea (35% to 48%) [45]. However, with additional sequence data this estimate will likely increase, as greater whole-genome coverage allows for *de novo *prediction of repetitive elements novel to NLL. LTR-retrotransposons were found to be the major component of repetitive DNA in NLL, similar to most higher plants characterised to date [13,45-48]. It appears that NLL has a relatively higher proportion of Ty1/Copia than Gypsy/DIRS1 repeat elements compared to soybean and pea [13,45], possibly reflecting a distinct evolutionary history specific to lupins and/or the genistoid clade.

The *de novo *sequencing of the NLL cv. Tanjil genome is underway and will involve sequencing 100 bp Illumina reads from a range of small (200 bp), medium (200-2000 bp) and large (5-40 kb) sized paired-end and mate-paired libraries. In addition to providing an initial genome survey and contributing new polymorphic markers, the BES dataset can be combined with these next-generation reads in the final genome assembly to connect scaffolds together across large assembly gaps. Where necessary, the BESs will also facilitate sequencing across gaps between scaffolds, as BAC clones corresponding to a BES in the vicinity of a gap can be isolated and sequenced individually.

The BESs described here are already being used to support the genome-wide identification of polymorphic genetic markers, such as SSRs with a total of 9966 SSR motifs identified. The analysis of the SSR profiles suggested some degree of consistency in the relative abundances of SSRs in NLL and other more characterised species (Figure 4). For example, consistent with *M. truncatula, L. japonicus, G. max, O. sativa *and *Arabidopsis*, class I di-nucleotide SSRs and class II hexa-nucleotide SSRs were the most abundant [41]. However, in NLL, the class I octa-nucleotide SSRs were also relatively overrepresented and penta-nucleotide SSRs were underrepresented. Conversely class II penta-nucleotide SSRs were relatively overrepresented and tri-nucleotide SSRs were underrepresented in NLL. The divergence of SSR abundance has also been documented for other types of SSRs [49,50].

The average density for the class I SSRs was approximately one SSR every 0.9 Kbp which is comparable to one SSR every 0.6-0.7 Kbp for the other five species compared. The average density for the class II SSRs however, was significantly less frequent (one every 51.0 Kbp) compared to the other five plant species (7.8-18.4 Kbp). This could be due to the relatively small NLL sample size or alternatively, the result may reflect the distinct phylogenetic placement of NLL compared to the other species.

Class I SSRs, which have longer repeat sequences and/or higher number of repeat units, are generally more mutable and thus more likely to be polymorphic between species than the SSRs with shorter repeat sequences and/or lower number of repeat units [51,52]. Forty eight candidate class I SSR markers were identified and 24 of these were initially screened for polymorphism between the parents of two RIL populations yielding nine and 14 novel SSR markers for the *cv*. Tanjil × *cv*. Unicrop and "wild" × "domestic" RIL populations, respectively. The degree of polymorphism in the initial set of class I SSRs between these four NLL lines shows that this is an effective way to identify and develop novel molecular markers. The majority of existing markers developed in NLL are AFLPs, MFLPs and RFLPs [19]. Besides these markers, 212 polymorphic PCR based markers are currently available in NLL and of these 212 PCR based markers, 39 are SSR markers [19,20]. None of the 212 PCR-based markers produced amplicons within the BESs presented in this publication by *in silico *PCR (amplicon size range of 20 bp to 10000 bp) [53]. The SSR markers presented in this study are therefore novel and are specific to regions of the NLL genome which have not been previously used for primer-based marker design. SSR markers are valuable tools that can be applied to the study of genetic diversity within collections, for example of *Lupinus *species and between cultivars [50] as well as refining existing genetic maps using high-throughput "multiplex-ready PCR", which has now been established for NLL. The SSR markers are also potentially useful for marker-assisted breeding across *Lupinus *species and other related species.

Phylogenetic analysis of the NLL BESs indicated only a small proportion (18.3%) of NLL BESs matched to the NCBI Nucleotide database and the majority (84%) of these were assigned to the species of the subfamily Papilionoideae of the Leguminosae. A large proportion of BESs (ca. 79.2%) did not match to any available legume nucleotide sequences despite almost full genome sequences being available for three species (*M. truncatula, L. japonicus *and *G. max*). This result suggests that there is significant genomic disparity between the Genistoid clade and other clades of the Papilionoideae subfamily. This further illustrates how selective sequencing of certain taxons can create biases in bioinformatic analysis and highlights the importance of exploring the NLL genome and the development of its genomic resources. The NLL BESs generated in this study represent the first genome-wide dataset for the genus *Lupinus *and provide an excellent foundation to further understand the evolution of the Leguminosae family.

## Conclusions

NLL is emerging as an important crop for agriculture and human health. As genetic and genomic studies in NLL within the genus *Lupinus *have been limited, the BAC library, the BAC-end sequences, and the SSRs markers described in this study are additional genomic resources for the species. These resources are critical for the construction of high-density physical and genetic maps and are valuable resources for map-based cloning and functional analysis of traits in lupin. They will greatly facilitate development of molecular and genetic tools for identifying and characterising genes involved in lupin crop improvement and in exploiting the crop for human nutrition. In addition, these resources provide a framework for further comparative genomics between lupins and other legumes and ongoing efforts towards assembling the complete NLL genome using next-generation sequencing.

## Methods

### Plant material

For the construction of the BAC library, seeds of *Lupinus angustifolius *L., narrow-leafed lupin (NLL), *cv*. Tanjil were germinated on moisturised filter paper at room temperature for two days. Seeds were subsequently grown hydroponically in half-strength Hoagland solution in a growth room at 22°C over a 16 h/8 h day/night schedule. After ten days, leaves were collected, frozen in liquid nitrogen and stored at -80°C.

For the Multiplex-Ready PCR assays to determine SSR lengths in four NLL lines, seeds of *cv*. Tanjil, *cv*. Unicrop, 83A:476 ("domestic") and P27255 ("wild") were germinated on moisturised filter paper at room temperature for two days. Subsequently, the germinated seeds were transferred into pots and grown in a temperature controlled growth chamber over 16 h/8 h day/night schedule, using fluorescent light at 100 to 120 μE m^-2 ^s^-1 ^and a constant temperature of 22°C. Leaf material was harvested from two-week-old plants for DNA isolation.

### Construction of the BAC library

BAC library construction was performed at the Australian Centre for Plant Functional Genomics, University of Adelaide, South Australia. The procedure has been described in detail in Shi *et al*. [54] and high-molecular weight DNA preparations were generated according to Zhang *et al*. [55]. Leaf tissue was ground in liquid nitrogen and the powder combined with homogenisation buffer. The homogenised sample was mixed with an equal volume of pre-warmed 1% low melting point (LMP) agarose and cast into plugs using plug molds (Bio-Rad). Following lysis, the agarose plugs were cut and digested with the restriction enzyme *Bam*HI. The DNA slices were size fractionated using a CHEF Mapper XA pulse-Field gel electrophoresis (PFGE) system (Bio-Rad), for 18 h at 11°C and 6 V/cm, using a 1-40 s pulse time and 120° field angle.

The 100-250 kb DNA fractions were excised and the DNA eluted in a Bio-Rad electro-eluter (Model 422), applying 10 mA per tube. Size-fractionated DNA was ligated to *Bam*HI Cloning-Ready pIndigoBAC-5 vector DNA (Epicenter) and transformed into ElectroMAX *E. coli *DH10B competent cells (Invitrogen). The transformation was carried out using a Bio-Rad Gene Pulser Xcell and a cuvette with a 1 mm gap at 1800 volts (Bio-Rad). Transformed cells were incubated at 37°C for 1 h in 1 mL LB, plated on to LB agar medium containing 12.5 μg/mL chloramphenicol and grown overnight at 37°C. Colonies were picked into wells of 384-well plates containing 70 μL LB freezing medium using a VersArray Colony Picker and Array System robot (Bio-Rad). Plates were incubated overnight at 37°C and used to make three copies of the library. Libraries were stored at -80°C. To print filters for hybridisation screening, clones from one copy of the library were arrayed in duplicate onto 22 cm × 22 cm positively charged nylon membranes (Amersham Hybond-N+ from GE Healthcare, or Performa II from Genetix) using a Qpix2 robot (Genetix). The three copies of the library were later transferred to and stored at CSIRO, Floreat, Western Australia.

Individual BAC clones were grown overnight at 37°C with vigorous shaking in 5-10 mL LB containing 12.5 μg/mL chloramphenicol, and the cultures were used to prepare BAC DNA by alkaline lysis. DNA of each clone was digested with 0.2 unit *Not*I restriction enzyme (New England BioLabs) and subjected to PFGE in 1% agarose gels and 1 × TAE buffer, for 18 h at 11°C and 6 V/cm, using a 1-40 s pulse time and 120° field angle. Fragments were photographed under UV light after ethidium bromide staining and insert sizes estimated by comparison to a Lambda Ladder PFG Marker (New England BioLabs).

### BAC library screening

A cDNA probe from the NLL β-conglutin gene (Beta2; [38]) was used for screening the BAC library. The probe was derived from the plasmid DNA containing the cDNA fragment of NLL Beta2 by PCR using M13 forward (5'-GTTGTAAAACGACGGCCAGT-3') and reverse (5'-CAGGAAACAGCTATGACC-3') primers. After purification with the Wizard SV 96 PCR Clean-up System (Promega), about 600 ng of the PCR product was labelled with [α-^32^P]-dCTP using Ready-To-Go DNA labelling beads (-dCTP) (Amersham Biosciences, Uppsala, Sweden) following the supplier's protocol. After incubating for 30 min at 37°C, the unincorporated nucleotides were removed using Illutra ProbeQuant G-50 Micro Columns (GE Healthcare).

Hybridisation was carried out at 68°C for 2 hr in ExpressHyb Hybridisation Solution (Clontech) following the supplier's protocol. The membranes were washed in 2 × SSC for 30 min and 1 × SSC for 1.5 hours. Subsequently, the membranes were exposed to an Imaging Screen K (Bio-Rad) for 30 min and the images captured using the Molecular Imager FX System and Quantity One software (Bio-Rad).

### Characterisation of BAC clones

Twelve clones were randomly selected from those which showed strong hybridisation signals. These clones were used in PCR reactions with a pair of primers designed from the conserved regions of the EST sequences of the seven β-conglutin genes [38]. The primer sequences were: forward 5'-TCCTCGTTGTACTCAATGGT-3' and reverse 5'-GGTTAAGGATATAAGAAGT-3' and the presence of β-conglutin sequences confirmed.

Eight BAC clones were selected for further characterisation. PCR reactions using primers specific for each β-conglutin gene and annealing temperatures (as described in [38]) were carried out with each BAC clone. PCR products were separated by gel electrophoresis and reactions which yielded a band sequenced using the PCR reaction primers. Where two bands were seen from one PCR reaction, each band was excised and purified from the agarose gel using the Qiaquick Kit (Qiagen) following the manufacturer's instructions. Retrieved sequence data was used to identify the best aligned NLL β-conglutin by BLAST analysis.

### BAC-end sequencing

The BAC-end sequencing was carried out by the Istituto di Genomica Applicata, Italy. BAC DNA was prepared from randomly selected clones using the MultiScreen Plasmid384 Miniprep Clearing plates (Millipore) coupled with a Biomek FX robot (Beckman). Sequencing of BAC-ends used an ABI3730xl platform (Applied Biosystems) with M13 forward and M13 reverse primers with Big-Dye Terminator chemistry (Applied Biosystems).

### Sequence cleaning and trimming

BAC-end sequences (BESs) were trimmed and filtered for quality and sequence contamination using seqclean (http://compbio.dfci.harvard.edu/tgi/software/), which had been modified to use the same BLAST parameters as NCBI VecScreen (http://www.ncbi.nlm.nih.gov/VecScreen/VecScreen.html). BESs were screened against contaminant databases including the UniVec vector database (Build #5.2 - Dec 7, 2009, ftp://ftp.ncbi.nih.gov/pub/UniVec), *E. coli *BL21 (DE3) complete genome sequence [NCBI NUC: CP001665.1] and plant organelle genomes (all NCBI mitochondrial, chloroplast and plastid genome sequences under the taxon "Eudicotyledons", [NCBI taxid: 71240]).

### Estimation of genome statistics, gene content and function

G:C content of BESs was calculated using custom perl scripts. Repetitive DNA content was estimated using RepeatMasker (version 3.2.9, http://www.repeatmasker.org) [56], with the REPBASE repeat database (version 20090604) [57]. RepeatMasker parameters were set to slow/sensitive alignment and restricted REPBASE entries to those of species within the taxon "Eudicotyledons" [NCBI taxid: 71240].

Trimmed NLL BESs were compared to the NCBI NR protein database by BLASTx [58]. BESs regions matching NCBI NR proteins and not matching to RepBase repeats [57] were assumed to contain protein-encoding genes. The proportion of filtered BLASTx-aligned BES regions relative to total BES sequence was used to estimate the percent of the NLL genome containing endogenous protein-encoding genes.

The functional content of the genome was estimated by running the 13985 trimmed BESs through the Blast2GO pipeline using default settings, blastx alignment to NR and ANNEX augmentation [59]. Gene Ontology summary PCA plots were generated via ReviGO [60]. REVIGO analysis focussed on functional annotations within the *Lupinus angustifolius *BES dataset only and did not involve multiple species comparisons. Assigned GOs were summarised via the 'plant' GOSlim subset (http://www.geneontology.org/GO_slims/goslim_plant.obo). Unfiltered GO terms were also summarised by conversion to MIPS FunCAT terms (http://www.geneontology.org/external2go/mips2go).

### Comparative genomics between NLL and other legumes

BESs were aligned via BLASTn [39] to the NCBI Nucleotide database. Phylogenetic distribution BLAST hits was visualised with MEGAN according to the lowest common ancestor of BLASTn BES hits [40]. The phylogenetic relationships in the cladogram presented in Figure 3 were derived from the NCBI taxonomy database.

### Screening of BES regions against published markers

Previous studies had published a total of 1104 markers used for the genetic mapping of NLL (summarised in Additional File 10). Of these 892 were PCR primer-based, comprising 74 AFLP, 646 MFLP, 159 RFLP, 6 isosyme and 7 phenotypic markers. The remaining 212 were tested for PCR amplification of BES sequence regions *in-silico *between a range of 20 to 10000 bp [53].

### Identification of SSRs and design of SSR-flanking primers

For the purposes of comparison with previous studies of SSRs in legumes, NLL SSRs were predicted via tandem repeats finder [61] according to the criteria outlined by Mun *et al*. [41]. SSRs were required to have a total length of ≥ 12 bp, SSR unit length ≥ 1-8 bp and 100% identical repetition of the SSR unit. Predicted SSRs were also distinguished into two classes according to total SSR length as per Mun *et al*. [41]: class I: SSR length ≥ 20 bp, class II: 12-19 bp. For the purpose of polymorphic marker discovery, additional Class I SSRs were predicted using less stringent criteria: allowing for a SSR unit size of 2-5 bp, a minimum of 75% identity to the SSR unit and a minimum number of five repetitions.

SSR-flanking primers were designed using primer3 [42]. Primer design was optimised for pairs producing 250 bp amplicons, primer lengths of 22 bp, melting temperatures (Tm) of 60°C, maximum difference in forward and reverse Tm of 1°C, maximum allowable primer hairpin lengths of 3 bp and maximum primer mononucleotide repeats of 2 bp. BESs were screened for potential repetitive DNA and organelle genome sequence contamination via sensitive RepeatMasker alignment to the Eudicotyledon subset of RepBase and BLASTn match (e-value < 1e-20) to NCBI Eudicotyledon organelle sequences. BAC-end sequences were also assembled via Cap3 [62] to identify potentially repeated regions (i.e. multi-gene families/uncharacterised repeats). Primer pairs designed within "repetitive" BESs were excluded from consideration as SSR marker candidates.

### Multiplex Ready PCR assays to determine SSR lengths in four NLL lines

DNA was isolated using the CTAB method [63] and dissolved in 10 mM Tris HCl (pH 8.0). The forward and reverse primer for 24 class I primer pairs were synthesised with the added nucleotide sequence 5'-ACGACGTTGTAAAA-3' and 5'-CATTAAGTTCCCATTA-3' respectively and called "locus specific primers". A list of the 24 class I SSR primer sequences is presented in Additional file 11. Two generic tag primers, *tagF *and *tagR *with the sequences 5'-ACGACGTTGTAAAA-3' and 5' CATTAAGTTCCCATTA 3', respectively, were also synthesised. The *tagF *primer was labelled at its 5'-end with one of the fluorescent dyes: VIC, FAM, NED and PET (Applied Biosystems). Multiplex-ready PCRs were subsequently carried out as described by Hayden *et al*. [64]. The multiplexed SSR PCR products were subjected to fragment analysis on an ABI3730 DNA analyser (Applied Biosystems) according to Hayden *et al*. [64] and SSR allele sizing used the Genemarker software (SoftGenetics LLC).

## Authors' contributions

LLG was involved in the construction, characterisation and screening of the BAC library and BAC-end sequencing. JKH carried out the bioinformatic analysis of BESs and designed the SSR markers. LGK set up the multiplex-ready PCR technique and tested the SSR markers. RF was involved in the isolation, sequencing and analysis of the β-conglutin clones. BJS was involved in the construction of the BAC library. KBS, CAA and LLG helped design the study. LLG drafted the manuscript with help from JKH, LGK, RF and KBS and all authors discussed results, commented on and approved the final manuscript.

## Supplementary Material

Additional file 1 **Sequences**. Alignment of the truncated sequences of the eight β-conglutin genes of *L. angustifolius*. Click here for file

Additional file 2 **Blast2GO**. RAW Blast2GO gene ontology assignments to BAC-end sequences of *L. angustifolius*. Click here for file

Additional file 3 **PlantGOSlim**. Summary of GO term abundance in the 13985 trimmed *L. angustifolius *BAC-end sequences, filtered by the 'plant' GOSlim subset. Click here for file

Additional file 4 **MIPS2GO**. Details of GO term conversions into MIPS FunCAT IDS. Click here for file

Additional file 5 **MIPS_FunCAT_full**. Summary MIPS FUNCAT annotations: BES counts are cumulative but BESs are not counted twice. Counts of offspring terms contribute to the counts of parent terms. Percentages are given in terms of the total 13985 tested. Click here for file

Additional file 6 **Summary of Gene Functions**. Summary of the relative proportions of predicted gene functions in *L. angustifolius*, summarised using the first two ranks of the MIPS FunCAT classification system. Click here for file

Additional file 7 **Summary of SSR motifs**. Simple Sequence Repeats (SSRs) identified in *L. angustifolius*. Click here for file

Additional file 8 **SSR primers**. Simple Sequence Repeats (SSRs) identified in *L. angustifolius*. Class I > = 20 bp, Class II 12-19 bp; fwd: forward; rev: reverse. Click here for file

Additional file 9 **Additional SSRs**. Additional SSRs predicated using tandem repeats finder (Benson 1999) and less stringent minimum SSR identity (> 75%). Click here for file

Additional file 10 **Markers used for the genetic mapping of NLL**. Summary of 1104 markers used for the genetic mapping of NLL Click here for file

Additional file 11 **Primer sequences tested**. Primer sequences for the 24 tested Class I SSR markers with additional adapter sequence (underlined). Click here for file
