# Supplementary figures and images for: Development of genomic resources for the narrow-leafed lupin (Lupinus angustifolius): construction of a bacterial artificial chromosome (BAC) library and BAC-end sequencing
Source: BMC Genomics. 2011 Oct 21;12:521. doi: 10.1186/1471-2164-12-521 (PMC3206524; doi:10.1186/1471-2164-12-521)

Additional File 1


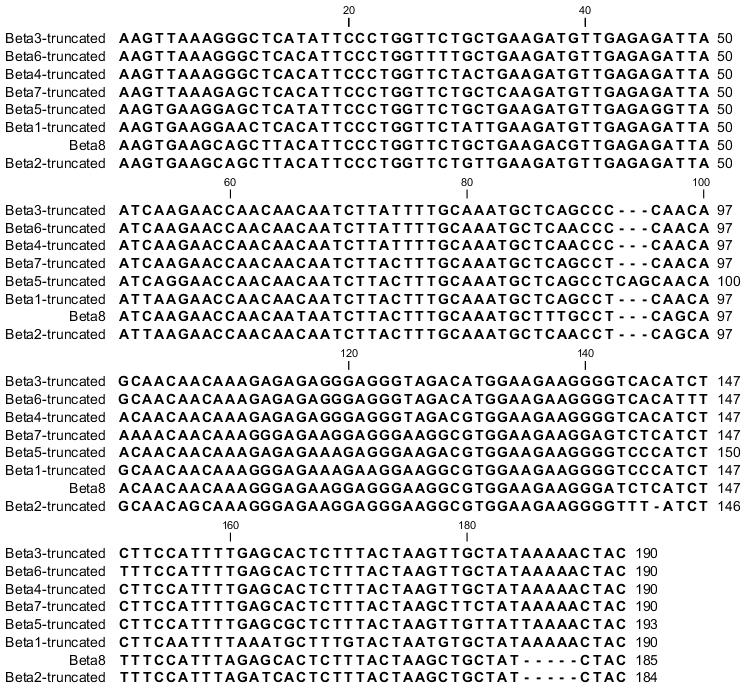

Supplement: Additional file 1 — Sequences. Alignment of the truncated sequences of the eight β-conglutin genes of L. angustifolius. [file 1471-2164-12-521-S1.DOC]
