# Supplementary material for: Development of genomic resources for the narrow-leafed lupin (Lupinus angustifolius): construction of a bacterial artificial chromosome (BAC) library and BAC-end sequencing
Source: BMC Genomics. 2011 Oct 21;12:521. doi: 10.1186/1471-2164-12-521 (PMC3206524; doi:10.1186/1471-2164-12-521)
Supplement: Additional file 6 — Summary of Gene Functions. Summary of the relative proportions of predicted gene functions in L. angustifolius, summarised using the first two ranks of the MIPS FunCAT classification system. [file 1471-2164-12-521-S6.DOC]

Table 2 Summary of the relative proportions of predicted gene functions in *L. angustifolius*, summarised using the first two ranks of the MIPS FunCAT classification system

| **FunCAT ID** | **Function** | **#BES** ^*^ | **% Gene content** ^§^ |
| --- | --- | --- | --- |
| **01** | **METABOLISM** | **534** | **3.82%** |
| 01.01 | amino acid metabolism | 263 | 1.88% |
| 01.02 | nitrogen and sulfur metabolism | 16 | 0.11% |
| 01.03 | nucleotide metabolism | 16 | 0.11% |
| 01.05 | C-compound and carbohydrate metabolism | 134 | 0.96% |
| 01.06 | lipid, fatty acid and isoprenoid metabolism | 144 | 1.03% |
| 01.20 | secondary metabolism | 32 | 0.23% |
| **02** | **ENERGY** | **47** | **0.34%** |
| 02.01 | glycolysis and gluconeogenesis | 48 | 0.34% |
| 02.04 | glyoxylate cycle | 1 | 0.01% |
| 02.07 | pentose-phosphate pathway | 9 | 0.06% |
| 02.08 | pyruvate dehydrogenase complex | 4 | 0.03% |
| 02.10 | tricarboxylic-acid pathway (citrate cycle, Krebs cycle, TCA cycle) | 3 | 0.02% |
| 02.25 | oxidation of fatty acids | 1 | 0.01% |
| 02.30 | photosynthesis | 5 | 0.04% |
| **10** | **CELL CYCLE AND DNA PROCESSING** | **2296** | **16.42%** |
| 10.01 | DNA processing | 901 | 6.44% |
| 10.03 | cell cycle | 6 | 0.04% |
| 11 | TRANSCRIPTION | 73 | 0.52% |
| 11.02 | RNA synthesis | 58 | 0.41% |
| 11.04 | RNA processing | 8 | 0.06% |
| **12** | **PROTEIN SYNTHESIS** | **16** | **0.11%** |
| 12.04 | translation | 1 | 0.01% |
| 12.10 | aminoacyl-tRNA-synthetases | 1 | 0.01% |
| **14** | **PROTEIN FATE (folding, modification, destination)** | **804** | **5.75%** |
| 14.01 | protein folding and stabilization | 20 | 0.14% |
| 14.04 | protein targeting, sorting and translocation | 6 | 0.04% |
| 14.07 | protein modification | 172 | 1.23% |
| 14.10 | assembly of protein complexes | 1 | 0.01% |
| 14.13 | protein degradation | 1 | 0.01% |
| **16** | **PROTEIN WITH BINDING FUNCTION OR COFACTOR REQUIREMENT (structural or catalytic)** | **2101** | **15.02%** |
| 16.01 | protein binding | 131 | 0.94% |
| 16.03 | nucleic acid binding | 1301 | 9.30% |
| 16.06 | motor protein | 4 | 0.03% |
| 16.07 | structural protein | 2 | 0.01% |
| 16.11 | amino acid binding | 4 | 0.03% |
| 16.13 | C-compound binding | 11 | 0.08% |
| 16.17 | metal binding | 280 | 2.00% |
| 16.19 | nucleotide binding | 325 | 2.32% |
| 16.21 | complex cofactor/cosubstrate binding | 43 | 0.31% |
| **18** | **PROTEIN ACTIVITY REGULATION** | **141** | **1.01%** |
| 18.01 | mechanism of regulation | 134 | 0.96% |
| 18.02 | target of regulation | 7 | 0.05% |
| **20** | **CELLULAR TRANSPORT, TRANSPORT FACILITATION AND TRANSPORT ROUTES** | **157** | **1.12%** |
| 20.01 | transported compounds (substrates) | 73 | 0.52% |
| 20.03 | transport facilitation | 16 | 0.11% |
| 20.09 | transport routes | 21 | 0.15% |
| **30** | **CELLULAR COMMUNICATION/SIGNAL TRANSDUCTION MECHANISM** | **138** | **0.99%** |
| 30.01 | intracellular signalling | 25 | 0.18% |
| 30.05 | transmembrane signal transduction | 45 | 0.32% |
| **32** | **CELL RESCUE, DEFENSE AND VIRULENCE** | **39** | **0.28%** |
| 32.01 | stress response | 32 | 0.23% |
| 32.07 | detoxification | 7 | 0.05% |
| **34** | **INTERACTION WITH THE CELLULAR ENVIRONMENT** | **29** | **0.21%** |
| 34.01 | ionic homeostasis | 2 | 0.01% |
| 34.03 | membrane excitability | 12 | 0.09% |
| 34.07 | cell adhesion | 2 | 0.01% |
| 34.11 | cellular sensing and response | 26 | 0.19% |
| **36** | **INTERACTION WITH THE ENVIRONMENT (Systemic)** | **34** | **0.24%** |
| 36.20 | plant / fungal specific systemic sensing and response | 26 | 0.19% |
| 36.25 | animal specific systemic sensing and response | 8 | 0.06% |
| **40** | **CELL FATE** | **19** | **0.14%** |
| 40.01 | cell growth / morphogenesis | 2 | 0.01% |
| 40.02 | cell differentiation | 3 | 0.02% |
| 40.10 | cell death | 15 | 0.11% |

| **41** | **DEVELOPMENT (Systemic)** | **24** | **0.17%** |
| --- | --- | --- | --- |
| 41.03 | plant development | 24 | 0.17% |
| **42** | **BIOGENESIS OF CELLULAR COMPONENTS** | **43** | **0.31%** |
| 42.01 | cell wall | 14 | 0.10% |
| 42.04 | cytoskeleton | 3 | 0.02% |
| 42.10 | nucleus | 1 | 0.01% |
| 42.25 | vacuole or lysosome | 2 | 0.01% |
| 42.26 | plastid | 4 | 0.03% |
| 42.27 | extracellular / secretion proteins | 16 | 0.11% |
| 42.33 | pilus/fimbria | 1 | 0.01% |
| 42.34 | prokaryotic cell envelope structures | 2 | 0.01% |
| **43** | **CELL TYPE DIFFERENTIATION** | **4** | **0.03%** |
| 43.02 | plant cell type differentiation | 4 | 0.03% |
| **45** | **TISSUE DIFFERENTIATION** | **2** | **0.01%** |
| 45.02 | plant tissue | 2 | 0.01% |
| **47** | **ORGAN DIFFERENTIATION** | **32** | **0.23%** |
| 47.02 | plant organ | 32 | 0.23% |
| **70** | **SUBCELLULAR LOCALIZATION** | **666** | **4.76%** |
| 70.01 | cell wall | 16 | 0.11% |
| 70.03 | cytoplasm | 45 | 0.32% |
| 70.04 | cytoskeleton | 2 | 0.01% |
| 70.07 | endoplasmic reticulum | 17 | 0.12% |
| 70.08 | Golgi | 16 | 0.11% |
| 70.10 | nucleus | 131 | 0.94% |
| 70.16 | mitochondrion | 192 | 1.37% |
| 70.19 | peroxisome | 13 | 0.09% |
| 70.22 | endosome | 5 | 0.04% |
| 70.25 | vacuole or lysosome | 26 | 0.19% |
| 70.26 | plastid *(includes chloroplast)* | 199 | 1.42% |
| 70.27 | extracellular / secretion proteins | 9 | 0.06% |
| 70.34 | prokaryotic cell envelope component | 8 | 0.06% |

Table 2 continue

**^*^ BES counts are hierarchically inclusive. BES counts of child FunCAT terms contribute to the counts of parent terms. BESs are counted only once in parent counts - BESs can potentially be assigned multiple terms therefore parent counts may not equal the sum of their children.**

^§^ **Estimated percentage of gene-content is calculated using the BES count divided by 13985 (the number of BESs analysed).**
